# Multidisciplinary approach to management of maternal asthma (MAMMA [copyright]): the PROTOCOL for a randomized controlled trial

**DOI:** 10.1186/1471-2458-12-1094

**Published:** 2012-12-19

**Authors:** Angelina Lim, Kay Stewart, Michael J Abramson, Susan P Walker, Johnson George

**Affiliations:** 1 Centre for Medicine Use and Safety, Faculty of Pharmacy and Pharmaceutical Sciences, Monash University, Parkville, VIC, Australia; 2 Department of Epidemiology and Preventive Medicine, Monash University, The Alfred Hospital, Melbourne, VIC, Australia; 3 Department of Perinatal Medicine, Mercy Hospital for Women, Melbourne, Victoria, Australia and University of Melbourne, Victoria, Australia

**Keywords:** Asthma, Pregnancy, Inhaled corticosteroids, Randomized controlled trial, Antenatal care, Intervention, Lung function tests, Multidisciplinary care

## Abstract

**Background:**

Uncontrolled asthma during pregnancy is associated with the maternal hazards of disease exacerbation, and perinatal hazards including intrauterine growth restriction and preterm birth. Interventions directed at achieving better asthma control during pregnancy should be considered a high priority in order to optimise both maternal and perinatal outcomes. Poor compliance with prescribed asthma medications during pregnancy and suboptimal prescribing patterns to pregnant women have both been shown to be contributing factors that jeopardise asthma control. The aim is to design and evaluate an intervention involving multidisciplinary care for women experiencing asthma in pregnancy.

**Methods/design:**

A pilot single-blinded parallel-group randomized controlled trial testing a Multidisciplinary Approach to Management of Maternal Asthma (MAMMA©) which involves education and regular monitoring. Pregnant women with asthma will be recruited from antenatal clinics in Victoria, Australia. Recruited participants, stratified by disease severity, will be allocated to the intervention or the usual care group in a 1:1 ratio. Both groups will be followed prospectively throughout pregnancy and outcomes will be compared between groups at three and six months after recruitment to evaluate the effectiveness of this intervention. Outcome measures include Asthma Control Questionnaire (ACQ) scores, oral corticosteroid use, asthma exacerbations and asthma related hospital admissions, and days off work, preventer to reliever ratio, along with pregnancy and neonatal adverse events at delivery. The use of FEV_1_/FEV_6_ will be also investigated during this trial as a marker for asthma control.

**Discussion:**

If successful, this model of care could be widely implemented in clinical practice and justify more funding for support services and resources for these women. This intervention will also promote awareness of the risks of poorly controlled asthma and the need for a collaborative, multidisciplinary approach to asthma management during pregnancy. This is also the first study to investigate the use of FEV_1_/FEV_6_ as a marker for asthma control during pregnancy.

**Trial registration:**

Australian New Zealand Clinical Trials Registry (ACTRN12612000681853)

## Background

Preterm birth and intrauterine growth restriction remain leading contributors to perinatal mortality and morbidity. Preterm birth is the leading cause of neonatal death, and over half of term stillbirths are associated with impaired fetal growth. Beyond the perinatal period, survivors of preterm birth and fetal growth restriction face a range of long term adverse health outcomes through infancy and childhood [1], and many adult diseases are now recognised to have their origins in fetal life [2-4]. Accordingly, continued efforts are necessary to identify interventions that may reduce the burden of preterm birth, and improve in utero fetal growth.

Poorly controlled asthma during pregnancy has been shown to be associated with an increased risk of preterm birth, low birth weight, and pre-eclampsia [5,6]. This data suggests that improved asthma control may be a means of reducing these important perinatal outcomes and that proper asthma management among pregnant women should be regarded as a health priority. In general, they should be managed in the same way as non-pregnant women with asthma, with the exception their asthma should be monitored at least monthly, as pregnancy can have a significant effect on asthma control [7,8]. A lack of knowledge amongst women regarding the risks of uncontrolled asthma during pregnancy is evident. Furthermore, there is a lack of confidence amongst health professionals when deciding the best management strategy for these women [9]. These concerns need to be addressed, as a starting point to optimise asthma control during pregnancy. Strategies to improve asthma management during pregnancy are warranted.

Preventive asthma medications at regular doses have been shown to be safe to use during pregnancy and the risks of reduction or discontinuation of these medications are far worse [10,11]. Asthma guidelines around the world strongly recommend that women continue their asthma medications during pregnancy to maintain adequate control [7,8,12-18]. However, women are still choosing to cease their asthma medications during pregnancy, many without consulting their doctors [19-21]. Reasons for this include concern over using any medication use during pregnancy, a desire for alternative therapies, perceptions of negative outcomes associated with steroid use, lack of support and guidance from health professionals regarding what to do with their asthma medications and the risks of poorly controlled asthma during pregnancy [22]. Moreover, women overestimate the teratogenic risks of asthma medication especially the steroid medications, with one report citing women perceived a 42% teratogenic risk for oral corticosteroid versus 12% risk for inhaled corticosteroid [23].

Prescribers have also been shown to be hesitant to prescribe and encourage use of asthma medications during pregnancy. Over a quarter of family physicians have said they would instruct their pregnant patients to decrease or discontinue asthma medication during pregnancy, when asthma was well controlled by current therapy [9], potentially jeopardizing asthma control. Pregnant women are also less likely to be treated with systemic corticosteroids for acute asthma exacerbations than non-pregnant women (50.8% versus 72.4%) [24].

The uncertainty and anxiety surrounding medication use and asthma control during pregnancy emphasise the crucial role of doctors, pharmacists and midwives in ensuring patient adherence to asthma medications during pregnancy and educating them on the risks of uncontrolled asthma during pregnancy. A collaborative approach between the pregnant women, doctors, midwives and pharmacists is needed to maintain adequate asthma control. Monthly monitoring has been recommended by guidelines [25] to maintain optimal asthma control as different stages of pregnancy can have an effect on asthma control [8]. We aim to test an intervention that allows for regular patient self-monitoring and a multidisciplinary health professional approach for asthma management during pregnancy; if successful it could justify funding for more support services for these women.

There also needs to be more detailed guidelines and objective measures for monitoring lung function for the treatment of pregnant women with asthma. The exhaled fraction of nitric oxide (FeNO) has been investigated as a marker for asthma control during pregnancy, but is expensive and not easily accessible [26]. Forced Expiratory Volumes in one and six seconds (FEV_1_/FEV_6_) has shown to be effective in detecting airway obstruction in the elderly and could be helpful in pregnancy [27]. FEV_1_/FEV_6_ may be a way of differentiating the shortness of breath associated with pregnancy from worsening asthma symptoms and a more convenient and affordable way of monitoring and guiding therapy in pregnant asthmatic women.

### Objective

To determine whether a multidisciplinary approach involving asthma education and regular monitoring during pregnancy will decrease asthma exacerbations with associated maternal and perinatal benefits. We hypothesis that the intervention group will have a better mean asthma control score than the control group at three and six months.

## Methods/design

### Study design

**Figure 1:**
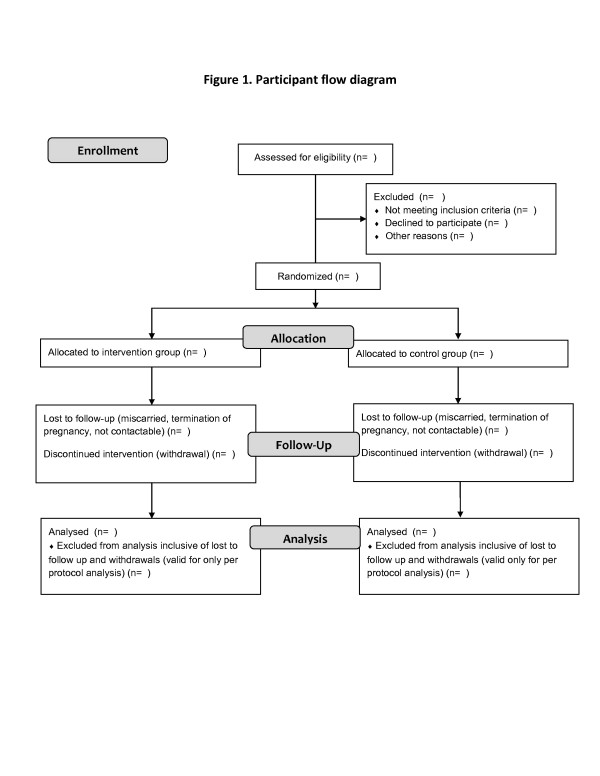
Participant flow diagram.

This is a single-blinded parallel-group randomized controlled pilot trial which will be conducted in the antenatal setting. It will test and evaluate a Multidisciplinary Approach to Management of Maternal Asthma (MAMMA©) which will involve education and regular monitoring by patients and their health professionals. The flow of the study design is outlined in Figure 1. Recruited participants, stratified by disease severity, will be allocated to the intervention or the usual care group in a 1:1 ratio. Both groups will be followed prospectively throughout pregnancy, and outcomes will be compared between groups at three and six months from baseline to evaluate the effectiveness of this intervention.

### Inclusion and exclusion criteria

All pregnant women with asthma attending antenatal outpatient clinics who are in their first or second trimester, and who can communicate in English will be considered. Patients who are under the age of 18 years or who have not had asthma symptoms (wheeze, chest tightness and/or use of their reliever asthma medication) in the last year will be excluded. Participants who are also unlikely to meet the demands of the trial will be excluded (e.g. planning to relocate during the trial). Furthermore, participants who were previously involved in our previous qualitative exploratory study conducted at the same maternity hospital, titled “Asthma during pregnancy; the experiences, concerns and views of pregnant women with asthma” [22] will also be excluded. Lastly, in the event of a miscarriage or termination of pregnancy, the participant will be excluded from the trial. Participant characteristics will be described in Table 1.

**Table 1:** Demographic and clinical characteristics of the study population at baseline

**Characteristic**	**Intervention group (n=30)**	**Control group (n=30)**
Patient information		
Age in years Mean [SD]		
Parity		
Gravidity		
Gestational age at first visit in weeks Mean [SD]		
Height in cm Mean [SD]		
Adherence Score Median [IQR]		
Ethnicity		
Australian		
Arabic		
Asian		
European		
Other		
Asthma severity^+^		
Intermittent to Mild		
Moderate to Severe		
Asthma medication		
SABA only		
ICS+SABA		
ICS/LABA + SABA		
FEV_1_ in litres Mean [SD]		
FEV_1_%predicted Mean [SD]		
FEV_1_/FEV_6_ Mean [SD]		
Co-morbidity		
Gestational diabetes		
Hypertension in pregnancy		
Anxiety/depression		
Other		
Smokers		
Health Care Concession Card holders		

ICS – inhaled corticosteroid, LABA- Long acting beta agonist, SABA – short acting beta agonist.

^+^Asthma severity classified by National Asthma Handbook [28].

Values are given as number (percentages) unless specified.

### Recruitment

Participants will be recruited from antenatal clinics of two major Victorian women’s hospitals. Four recruitment methods will be used to ensure the sample size is reached efficiently:

1. Pregnant women who have self reported asthma will be approached at their antenatal outpatient appointment.

2. Advertisement posters publicising the trial will be placed in the outpatient department alongside an ‘expression of interest’ box where participants can leave their contact details. Study packs (including an explanatory statement with an expression of interest form and a reply paid envelope) will also be available in the outpatient department so potential participants can take the information home to read and post back expression of interest forms.

3. Midwives will also be asked to help identify eligible pregnant women with asthma during their first outpatient visit. They will be asked to approach any woman who has indicated they are asthmatic and hand them a study pack, which will be available in the outpatient department.

4. A list of pregnant asthmatic women who have had their first outpatient visit will be generated weekly from the medical records database which stores antenatal information for all hospital patients. Study packs will be posted to the women who are on the weekly list.

Written informed consent will be obtained from all participants. All participants will be over the age of 18 years and will have competency to consent. During the recruitment phase, each participant will be asked to nominate her preferred family physician (general practitioner) to be involved in the trial who will be the lead clinician responsible for her asthma management during pregnancy.

### Group allocation

Participants will be asked basic questions to determine their asthma severity in accordance with the National Asthma Council Management Handbook [28] classifications. Participants will be stratified into two groups: mild intermittent asthmatics and moderate-severe persistent asthmatics. Within these two strata, block randomization using random blocks of four and six will be conducted using the sealed opaque envelope method. A random sequence of numbers will be generated using the Random allocation software program® by an external researcher who is not part of the research team. Only this researcher will be aware of the allocation sequence. Numbered envelopes will be opened by the leading investigator AL to allocate participants to the usual care group (UCG) or the multidisciplinary care group (MCG) at time of recruitment and will enrol the participant into the study. Stratification and block randomization are included to ensure a balance of asthma severities between groups and an even number of participants per group. Outcome assessors will be blinded to participant group allocation.

### Intervention (MCG participants)

**Figure 2:**
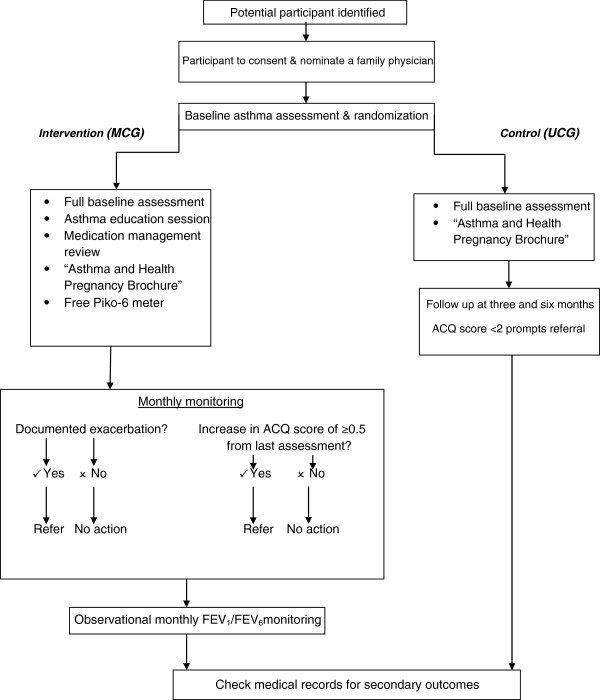
MAMMA© study design.

The Multidisciplinary Approach to Management of Maternal Asthma (MAMMA©) intervention will embrace a collaborative approach involving the participant’s family physician, pharmacist and asthma educator. Details of the intervention are described in Figure 2. Asthma education, monitoring, feedback and follow-up are integral components of the monthly intervention. Every month, participants in the intervention group will be contacted by the trial’s nominated pharmacist for an hourly session to assess their asthma control by administrating the Asthma Control Questionnaire (ACQ) [29] and a short data collection form which inquires about oral corticosteroid use, asthma related hospital admissions, days off work and preventer to reliever use ratio. The ACQ states that an increase in a score of 0.5 is a clinically significant deterioration of asthma control [29]. The trial pharmacist will provide feed back to the participant’s nominated family physician if the ACQ score has increased by 0.5 or greater and if there has been a documented exacerbation since the last monthly visit. The pharmacist and family physician will then collaborate on appropriate step up therapy for the participant. It is anticipated that this close monthly monitoring will maintain the participant’s asthma under closer control during pregnancy. Each participant in the MCG will be given a handheld device (PiKo-6) to use as they please for home monitoring of lung function and instruction in the use of PiKo-6. They will also receive pharmacist led medication management review at the beginning of the trial, periodic review of inhaler device technique by asthma educator, trigger avoidance and smoking cessation support (if relevant). The MCG will also have their FEV_1_/FEV_6_ measured monthly during the trial. Adherence and uptake of the intervention will be described in Table 2.

**Table 2:** Adherence to intervention by intervention and control groups

	**Intervention group n(%)**	**Control group n(%)**
No. of reviews recommended by intervention pharmacist		
No. of asthma action plans up taken		
No. of recommended medication changes up taken		

### Control (UCG participants)

The control group will receive usual medical care; this normally includes their regular antenatal visits ranging from weekly to monthly depending on trimester and other complications. They will not receive the intervention, any additional monitoring or education sessions like participants in the MCG. If during follow ups at three and six months, their asthma control becomes a concern (2 or more documented exacerbations without resolution (i.e. increasing preventer dose) since prior assessment or their ACQ score exceeds 2, the participant and their family physician will be notified (with participant permission). This notification will be taken into account when we do the analysis.

Both groups will be given a summarised version of the “Asthma and Healthy Pregnancy” brochure from the Asthma Foundation of New South Wales, Australia which is a pamphlet on basic asthma facts to avoid unfair disadvantage to participants in the control group and to minimise the risks of poorly controlled asthma.

#### Outcomes

The primary outcome of the trial will be the ACQ score. It is hypothesised that the MCG will have a higher average ACQ score than the UCG at 3 and 6 months. Secondary outcomes will include asthma-related hospital visits and days off work, oral corticosteroid use and preventer to reliever use ratio. Pregnancy outcomes to be collected will be the development of antenatal complications, such as hypertensive disorders of pregnancy, antepartum haemorrhage, gestational diabetes and gestational age at delivery. Neonatal outcomes will include gestational age and birth weight percentile, Appearance Pulse Grimace Activity and Respiratory (APGAR) scores, admission to neonatal intensive care or special care nursery, mode of delivery and any postnatal complications. The study is not powered to assess the effect of the intervention on pregnancy and neonatal outcomes; however, these outcomes will still be documented as there is an abundance of information available highlighting poorly controlled asthma during pregnancy leading to maternal and perinatal hazards. Results of outcomes will be described in Table 3.

**Table 3:** ACQ scores and asthma outcomes at Baseline, 3 and 6 months

	**Baseline**		**At 3 months**		**At 6 months**	
Result	Intervention group (n=30)	Control group (n=30)	Intervention group (n=30)	Control group (n=30)	Intervention group (n=30)	Control group (n=30)
ACQ score						
No. of asthma related hospital visits						
Days off work						
No of days of asthma related oral corticosteroid use						
Preventer to reliever ratio						

Values are given as mean (SD) or number (percentages).

### Follow up

ACQ scores, asthma-related hospital visits and days off work, oral corticosteroid use and preventer to reliever use ratios will be compared at three and six months between groups. Both groups will be assessed using the same data collection form at 3 and 6 months (once in their second trimester and once in their third trimester) and the results will be compared. The assessor collecting the data at three and six months in both groups will be different from the intervention pharmacist and will be blinded to participant group allocation. The assessor will not be given any clinical information about each participant and will be unaware of the study protocol (including details of the intervention) just in case a participant inadvertently discloses details to the assessor. Pregnancy and neonatal outcomes will be confirmed via medical records shortly after delivery.

### Sample size calculation

This is a pilot study, however sample size was still calculated to guide recruitment. Using a conservative standard deviation of 0.66, to detect a change in ACQ score of 0.5 or more between groups, [18] a sample size of 29 per arm would have 80% power with a two sided 5% significance level assuming the two variances are the same. To allow for 20% attrition, 35 participants will be recruited in each arm.

### Data analysis

The primary analysis will be intention to treat. Per protocol analyses will also be conducted. The baseline characteristics of participants in the intervention and control groups will be compared using Chi-square, Student *t*-test or Mann–Whitney tests if distributional assumptions are not satisfied. ACQ scores at baseline, three and six months will be compared using Mann–Whitney test. Secondary outcomes will be listed as descriptive statistics and analysed using similar tests. FEV_1_/FEV_6_ trends will be described as these are observational data only. Sensitivity and predictive validity of the ACQ would also be investigated.

### Ethics

This trial has been approved by the Mercy Health Research Ethics Committee, The Royal Women’s Hospital Research Ethics Committee and Monash University Human Research Ethics Committee. The trial has also been registered with the Australian and New Zealand Clinical Trial Registry ACTRN12612000681853.

## Discussion

The proposed intervention has the potential to improve health outcomes in pregnant women with asthma, by reducing the incidence and severity of maternal exacerbations, and potentially reducing the perinatal morbidity associated with preterm birth and impaired fetal growth. These interventions have the potential to reduce health care costs through fewer asthma-related unplanned medical and emergency department visits for pregnant women. In addition, the costs associated with poor pregnancy and neonatal outcomes that can result from poorly controlled asthma i.e. pre-term births and low birth weight babies (parenteral nutrition costs, Neonatal Intensive Care admissions, assisted ventilation etc.) would be reduced. The interventions could also have a positive impact on the health of future generations. If the proposed intervention is successful and cost-effective, it may justify additional support services for pregnant women with chronic health conditions such as asthma. These support services could readily be made available and accessible in the community as well as hospital settings.

The trial is also the first to investigate the use of FEV_1_/FEV_6_ monitoring during pregnancy. This trial is powered to assess the primary asthma symptom-related endpoint, but not sufficient enough to draw conclusions in regards to maternal and perinatal outcomes. It has been expressed that it is difficult to distinguish between the shortness of breath associated with pregnancy and the airway obstruction associated with asthma [22]. Observational findings from this trial could help examine patterns and trends of FEV_1_/FEV_6_ values. It is suggested that overweight individuals have a lower FEV_1_ and the expanding uterus during pregnancy may further reduce this by decreasing lung volumes [30]. However, significant changes in FEV_1_ coupled with reduced FEV_1_/FEV_6_ ratios are likely to be due to deteriorating asthma control.

Moreover, as the lung function of a pregnant woman differs from that of a non-pregnant woman [30], a target range for FEV_1_/FEV_6_ measurements during pregnancy could be identified and prompt further research in using this ratio as a marker for asthma control during pregnancy. FEV_1_/FEV_6_ could be a simple and easier method of assessing asthma control and help prescribers better distinguish between asthma symptoms and the shortness of breath associated with pregnancy. Women will have the convenient option of monitoring their asthma at home using a handheld spirometer and adjusting therapy or management according to an individualised asthma action plan.

## Conclusion

The MAMMA trial will investigate the role of participant self-monitoring and multidisciplinary team care in the management of asthma during pregnancy. The use of FEV_1_/FEV_6_ as a measure of lung function in pregnancy will also be explored. Empowering women to take control of this common chronic health condition and a multidisciplinary approach to management could potentially reduce the burden of asthma during pregnancy.

## Abbreviations

ACQ: Asthma Control Questionnaire; APGAR: Appearance, Pulse, Grimace, Activity, Respiratory; FEV_1_: Forced Expiratory Volume in one second; FEV_6_: Forced Expiratory Volume in six seconds; ICS: Inhaled Corticosteroid; MCG: Multi-disciplinary Care Group; UCG: Usual Care Group.

## Competing interests

Dr Johnson George and Prof Michael Abramson had an investigator-initiated research (IIR) grant from Pfizer for a separate research project which targets smoking cessation. All other authors declare no competing interests.

## Authors’ contributions

Angelina Lim (PhD candidate) developed and designed the trial with input from all the other authors (AL, JG, KS, MA and SW). Ms Lim wrote the first draft of the protocol and refined it based on comments and feedback from all the other authors. All authors read and approved the final manuscript.

## Authors’ information

AL is a PhD student at Monash University and works as a pharmacist in Mercy Hospital for Women. She also works part-time in community pharmacy and for the Asthma Foundation of Victoria. Her research is based on optimizing the management of asthma during pregnancy.

KS is an Associate Professor in the Centre for Medicine Use and Safety at Monash University. Her research interests include medication adherence and asthma management.

MJA is Professor of Clinical Epidemiology at Monash University and a Visiting Medical Officer in Allergy, Immunology & Respiratory Medicine at the Alfred Hospital in Melbourne.

SW is a Professor of Maternal Fetal Medicine, University of Melbourne, and Director of Perinatal Medicine, Mercy Hospital for Women

JG is a Senior Lecturer in the Centre for Medicine Use and Safety at Monash University. His research targets improving asthma and chronic obstructive pulmonary disorder management, medication adherence, smoking cessation and medication use in pregnancy.

## Pre-publication history

The pre-publication history for this paper can be accessed here:

http://www.biomedcentral.com/1471-2458/12/1094/prepub
